# Assessing the impact of bacterial heterogeneity on bacteriophage population dynamics

**DOI:** 10.1007/s10928-026-10025-y

**Published:** 2026-03-09

**Authors:** Massinissa Beldjenna, Jérémie Guedj, J. G. Coen van Hasselt, Tingjie Guo

**Affiliations:** 1 Leiden Academic Centre for Drug Research (LACDR), Leiden University, Leiden, Netherlands; 2 Inserm, IAME, Université Paris Cité, Paris, France

**Keywords:** Bacteriophage, Viral dynamics, Cellular variability, Parameter distribution, Multi-scale modeling, Intracellular replication, PK/PD modeling, Lokta-Volterra integro-differential equations

## Abstract

Bacteriophages follow complex replication dynamics in codependence with their host cells. The corresponding dynamics are usually described with three key parameters: the adsorption rate $\varphi$, the burst size $\beta$ and the latent period $\tau$. Single-cell experiments have shown cellular variability in latent period and burst size. Yet, those parameters are traditionally modelled as uniform values for the whole bacterial population, which may introduce bias in the predicted profiles. Here, we systematically assessed the relevance of modelling this cell-level variability in the context of bacteriophage dynamics. We developed a comprehensive modelling framework, aiming to: (i) identify which parameter distributions impact population dynamics, (ii) quantify how difference in distribution can influence predictions, and (iii) inform model selection by comparing relevant modelling approaches. A distributed delay differential equation model with randomly distributed latent period, burst size and adsorption rate within the cell population was developed. It also included logistic growth of the host cell, saturable adsorption rate and intracellular replication dynamics. This model was compared to classical models, both fixed delay differential equation and transit compartments models, over typical ranges of phage parameters. Only the distribution of the latent period and its influence on burst size impacted population dynamics. The two classical models proved to be good approximations of dynamics, but only at low cellular variability. As such, although the classical approaches can be preferred at lower variability, this distributed framework is warranted when high variability is expected, or when the other approaches fail to accurately capture the dynamics.

**Supplementary Information:**

The online version contains supplementary material available at 10.1007/s10928-026-10025-y.

## Introduction

Bacteriophages or phages have gained renewed interest in the past decade due to the increasing threat of multidrug-resistant bacterial infections and their potential utility as anti-infective therapy [1]. Bacteriophages are viruses which infect target bacteria and exhibit complex population dynamics in interaction with their hosts. In order to optimise bacteriophage therapy, we need to quantitatively understand and predict the effects of said interactions. To that end, mathematical models describing bacteria-phage interactions have been previously developed [2, 3]. These models typically consider three key parameters: (i) the rate at which phages bind to the target bacteria (adsorption rate $\varphi$); (ii) the delay between infection and lysis of the host bacterium (latent period $\tau$); and (iii) the number of new phages released per lysed bacterium (burst size $\beta$). While such models provide valuable insights, they generally neglect the intrinsic heterogeneity between individual bacteria, assuming uniform parameter values across the entire bacterial population [2].

**Fig. 1:**
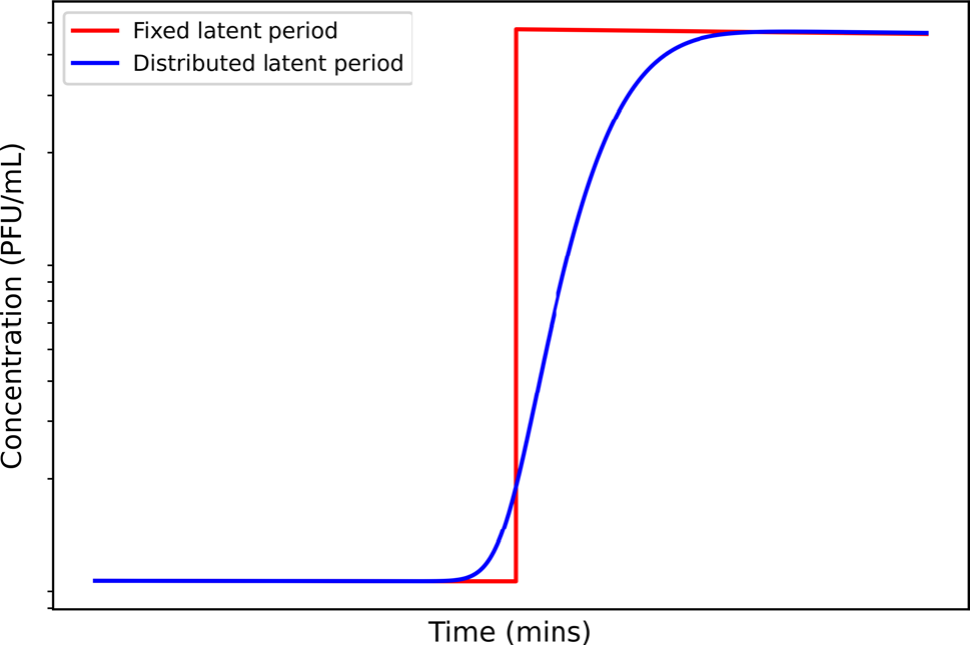
Single-step growth curves simulated with a fixed latent period (red curve) and a distributed latent period (blue curve)

Single-cell studies have demonstrated substantial variability in both latent period [4] and burst size [5] between individual bacteria. Moreover, experimental studies have shown that even under conditions of synchronised infection [6], phage concentrations increase gradually over time rather than abruptly [7], which is consistent with a distributed latent period (Fig. 1). Yet, the latent period is typically reported as a uniform value, when the first lysis is observed experimentally [8]. This approach not only disregards the parameter distribution, but also could lead to a systematic bias with regard to the latent period observed at the single-cell level [9].

The distributed nature of the latent period has been described using distributed delay differential equations (DDDE) [10, 11]. However, such models are rarely used in practice. Their limited adoption is largely due to their theoretical complexity, long computation times, and the scarcity of accurate experimental data on relevant parameter distributions. It remains unclear whether and under which conditions incorporating cellular variability meaningfully improves model predictions of population dynamics.

**Fig. 2:**
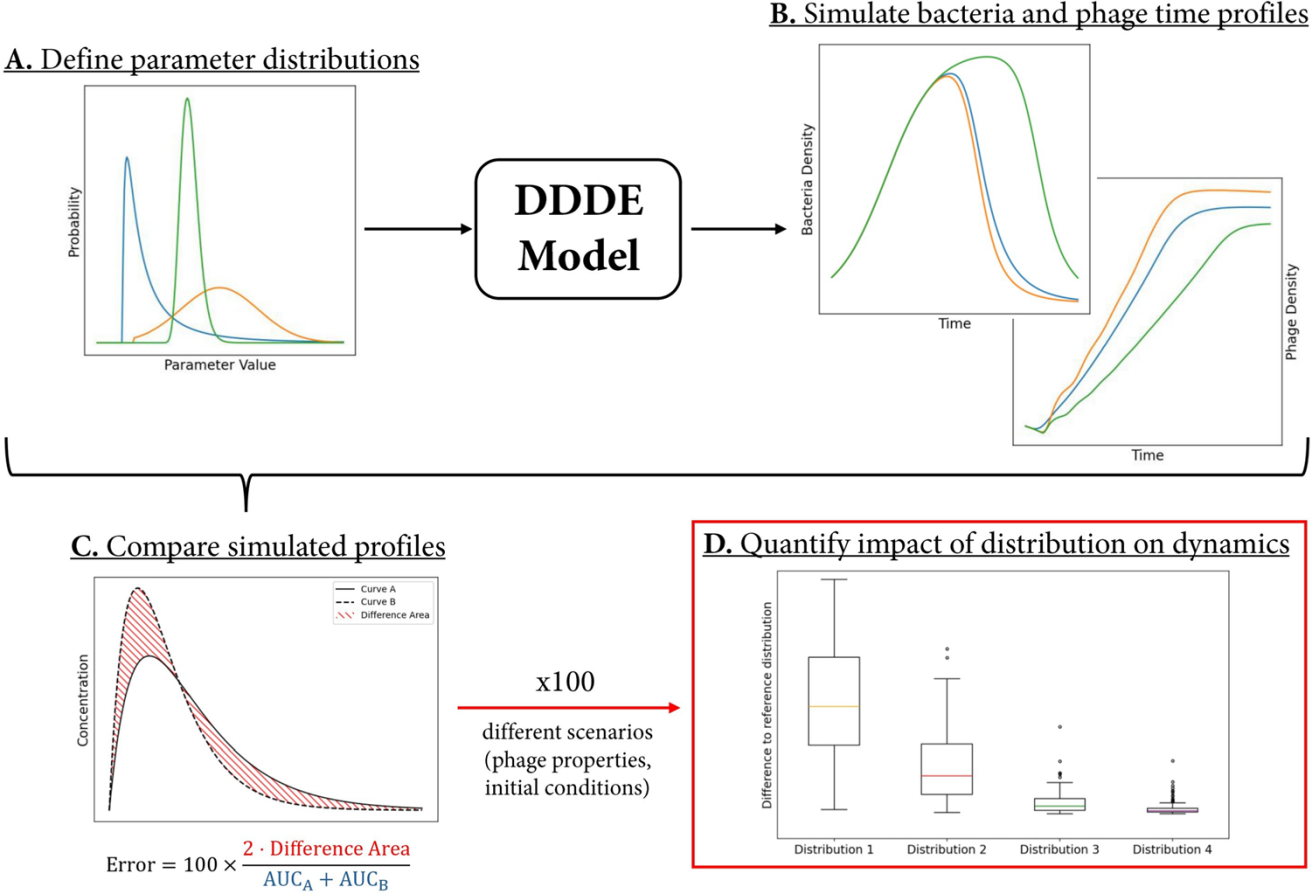
Workflow of the study: A. Definition of parameter distributions to compare (Section “Considered distributions”). B. Simulation of the corresponding bacteria and phage time profiles with a distributed delay differential equation (DDDE) model (Section “Distributed parameter framework”). C. Comparison of resulting bacterial and viral load profiles for different distributions (Section “Evaluation of the impact of the distribution”). D. Compilation of the differences observed after repeating steps A to C for 100 sets of parameters, to quantitatively evaluate the overall effect of parameter distribution on population dynamics (Section “Evaluation of the impact of the distribution”)

The current study aimed to address this gap by: i) identifying analytically parameter distributions which impact population dynamics; ii) evaluating the sensitivity of population dynamics to the shapes of these distributions; (iii) quantifying the impact of fixing the parameters to representative values on population dynamics; and (iv) informing model selection by comparing relevant modelling alternatives. To this end, we used a theoretical bottom-up modelling approach. We first developed a bacteriophage pharmacodynamic model accounting for cellular variability (Sections “Base model structure” & “Distributed parameter framework”). We then used this model to simulate bacteria and phage dynamics (Fig. 2B) for different parameter distributions (Section “Considered distributions”, Fig. 2A). We compared the obtained profiles and defined an error metric to quantify their differences (Fig. 2C). To systematically and quantitatively assess how the parameter distributions impact the dynamics, we repeated the previous steps with 100 different sets of model parameters (Section “Evaluation of the impact of the distribution”, Fig. 2D).

## Theoretical

### Base model structure

The study was based on a classic mathematical model for phage dynamics [2]:

1 $$\begin{aligned} \frac{dU}{dt}(t)&= r(t) \cdot U(t) - \delta _{U} \cdot U(t) \nonumber \\&\qquad \qquad \,\,\,- \varphi (t) \cdot U(t) \cdot P(t) \nonumber \\ \frac{dI}{dt}(t)&= \varphi (t) \cdot U(t) \cdot P(t) - \delta _I \cdot I(t) \\&\qquad \qquad \,\,\,- e^{- \delta _I \tau } \cdot \varphi (t-\tau ) \cdot U(t-\tau ) \cdot P(t-\tau ) \nonumber \\ \frac{dP}{dt}(t)&= \beta \cdot e^{- \delta _I \tau } \cdot \varphi (t-\tau ) \cdot U(t-\tau ) \cdot P(t-\tau ) \nonumber \\&\qquad \qquad \,\,\,- \delta _P \cdot P(t) - \varphi (t) \cdot (U(t) + I(t)) \cdot P(t)\nonumber \end{aligned}$$

where U, I and P are the concentrations of uninfected bacteria, infected bacteria, and phages respectively, $\delta _U, \delta _I, \delta _P$ are their respective decay rates, *r*(*t*) is the bacterial growth rate, $\varphi (t)$ is the adsorption rate, $\beta$ is the burst size and $\tau$ is the latent period. Here, phages may adsorb to either uninfected or infected bacteria with the adsorption rate $\varphi (t)$.

Equation 1 depends on the states of the variables at time $t-\tau$, which makes this a delay differential equations (DDE) model. Such equations require information on the history of the state variables. Here, t=0 is taken as the time of phage administration, hence:

2 $$\begin{aligned} \forall t < 0, P(t) = 0 \end{aligned}$$

Any history of $\varphi (t)$ and *U*(*t*) thus results in $\forall t < 0, \varphi (t) \cdot P(t) \cdot U(t) = 0$. This history of *P*, as well as usual initial conditions at t=0 for the state variables, is consequently enough to fully determine the system.

The bacterial growth was assumed logistic [12], which gives:

3 $$\begin{aligned} r(t) = r_{max} \cdot (1 - \frac{U(t) + I(t)}{K_C}) \end{aligned}$$

where $r_{max}$ is the max bacterial growth rate and $K_{C}$ is the ceiling concentration of bacterial growth.

A saturable adsorption rate was assumed as follows [13]:

4 $$\begin{aligned} \varphi (t) = \varphi _{max} \cdot \frac{P_{50}}{P_{50} + P(t)} \end{aligned}$$

where $\varphi _{max}$ is the maximal adsorption rate and $P_{50}$ is the phage concentration at which the adsorption rate is half its maximal value.

### Distributed parameter framework

To account for cellular variability, Eq. 1 was adapted with distributed parameters. Prior cell-studies showed evidence of dependence of burst size on latent period [14], which was accounted for by introducing conditional distributions.

The latent period $\tau$ is randomly distributed around a fixed mean value $\tau _0$. The corresponding probability density is denoted as $f_{\tau }(\tau )$. A minimal possible latent period $\tau _{min}$ can also be defined to account for the fact that a phage cannot instantly lyse a bacterium [10].

For a given value of $\tau$, the burst size $\beta$ is randomly distributed around a mean value $\beta _0(\tau )$, for which the conditional probability density is written as $f_{\beta |\tau }(\beta | \tau )$. The relationship between mean burst size $\beta _0(\tau )$ and latent period $\tau$ was described using the following phenomenological expression [14]:

5 $$\begin{aligned} \beta _0(\tau ) = \beta _{max} \frac{e^{r_\beta (\tau - D)} - 1}{e^{r_\beta (\beta \tau _{50} - D)} + e^{r_\beta (\tau - D)} - 2} \end{aligned}$$

where $\beta _{max}$ is the maximal burst size, $r_{\beta }$ the intracellular phage replication rate, $\beta \tau _{50}$ is the latent period corresponding to half of the maximum burst size and D is a time delay below which burst size is 0. A fixed value for $\beta _0(\tau )$, independent of $\tau$, was also explored (appendix).

The population mean burst size, $\beta _{0,mean} = \int _0^{+\infty } \beta _0(\tau )f_\tau (\tau )d\tau$, is the parameter typically determined from single-step growth curves [8]. Therefore, $\beta _{max}$ in Eq. 5 was re-expressed as a function of $\beta _{0,mean}$ (derivation in appendix):

6 $$\begin{aligned} \beta _{max} = \frac{\beta _{0, mean}}{\int _{\tau = \tau _{min}}^{+\infty } \frac{e^{r_\beta (\tau - D)} - 1}{e^{r_\beta (\beta \tau _{50} - D)} + e^{r_\beta (\tau - D)} - 2} \cdot f_\tau (\tau ) \cdot d\tau } \end{aligned}$$

The adsorption rate $\varphi (t)$ is randomly distributed around a mean value $\varphi _{0}(t)$ that is a function of time at which the phage adsorbs to the bacteria (assumed to follow Eq. 4). The corresponding conditional probability density is written $f_{\varphi |t}(\varphi |t)$.

The associated differential equations are:

7 $$\begin{aligned} \frac{dU}{dt}(t)&= r(t) \cdot U(t) \nonumber \\&\qquad \quad - \int _{\varphi = 0}^{+ \infty } \varphi \cdot U(t) \cdot P(t) \cdot f_{\varphi | t}(\varphi | t) \cdot d\varphi - \delta _{U} \cdot U(t)\nonumber \\ \frac{dI}{dt}(t) =&\int _{\varphi = 0}^{+ \infty } \varphi \cdot U(t) \cdot P(t) \cdot f_{\varphi | t}(\varphi | t) \cdot d\varphi \\&- \int _{\tau = 0}^{t} \int _{\varphi = 0}^{+ \infty } e^{- \delta _I \tau } \cdot \varphi \cdot U(t-\tau ) \cdot P(t-\tau ) \nonumber \\&\cdot f_{\varphi | t}(\varphi | t - \tau ) \cdot f_{\tau }(\tau ) \cdot d\varphi \cdot d\tau - \delta _I \cdot I(t)\nonumber \\ \frac{dP}{dt}(t) =&\int _{\tau = 0}^{t} \int _{\varphi = 0}^{+ \infty } \int _{\beta = 0}^{+ \infty } \beta \cdot e^{- \delta _I \tau } \cdot \varphi \cdot U(t-\tau ) \cdot P(t-\tau ) \nonumber \\&\qquad \quad \cdot f_{\beta | \tau }(\beta | \tau ) \cdot f_{\varphi | t}(\varphi | t-\tau ) \cdot f_{\tau }(\tau ) \cdot d\beta \cdot d\varphi \cdot d\tau \nonumber \\&- \int _{\varphi = 0}^{+ \infty } \varphi \cdot (U(t) + I(t)) \cdot P(t) \cdot f_{\varphi | t}(\varphi | t) d\varphi - \delta _P \cdot P(t)\nonumber \end{aligned}$$

**Fig. 3:**
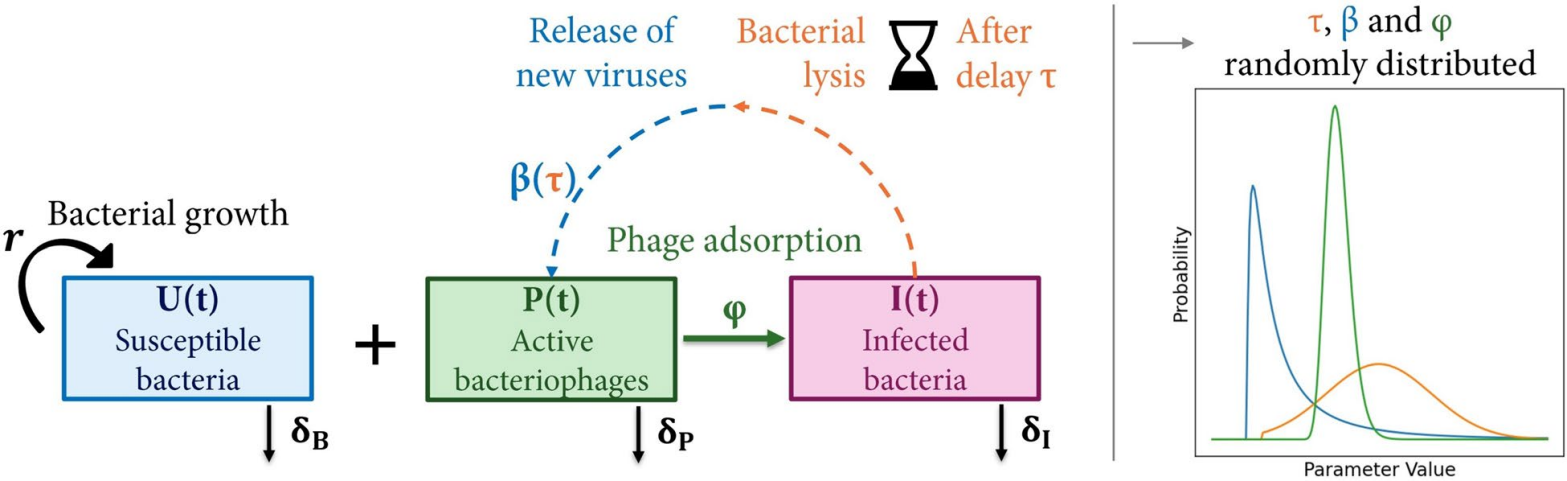
Compartmental representation of the distributed delay differential equations model with intracellular replication dynamics

This model (represented in Fig. 3) is a distributed delay differential equation (DDDE) model accounting for the dependence of $\beta$ on $\tau$ (Eq. 5), associated with intracellular replication dynamics. Those equations are a naive representation of what is referred to as the "Full DDDE" model. Similarly to the DDE model, Eq. 2 provides sufficient history for this model structure. In particular, it is not necessary to integrate for $\tau> t$ because $\forall \tau> t, P(t-\tau ) = 0.$

## Methods

### Evaluation of the impact of the distribution

#### Analytical assessment

An analytical analysis of the differential (Eq. 7) was first conducted to gain general insights on whether the distributions of the latent period $\tau$, the burst size $\beta$ and the adsorption rate $\varphi$ influence population dynamics.

The roles of the distributions of the burst size $\beta$ and the adsorption rate $\varphi$ in population dynamics were fully analytically determined. However, the analytical study was insufficient for the latent period $\tau$. Further simulation work was thus required. As such, the impact of the latent period distribution was investigated through both visual and quantitative analyses of simulated scenarios.

#### Exploratory analysis

Time profiles of bacteria and bacteriophages were simulated with different latent period distributions. Those profiles were used to inspect how the selection of the distribution model influenced the shape of the simulated curves. In the exploratory study, a visual of the profiles was repeated for various sets of parameters. An example of profiles was included in the results section.

#### Quantitative analysis

The difference between two simulated profiles A and B was quantified with a metric based on the normalised area between the curves (Fig. 2C):

8 $$\begin{aligned} \text {Error} (\%) = 100 \cdot \frac{2 \cdot \text {Difference Area}}{AUC_A + AUC_B} = 100 \cdot \frac{2 \cdot \int _{t=0}^{+\infty } |X_A - X_B| \textit{ dt}}{\int _{t=0}^{+\infty } X_A \textit{ dt} + \int _{t=0}^{+\infty } X_B \textit{ dt}} \end{aligned}$$

Given any two distribution models, a Monte Carlo analysis of the difference between the two models for both bacteria and phage profiles was conducted. 500 sets of parameters were randomly generated, with $r_{max}, \delta _U, \delta _I, \delta _P, \tau _0, \tau _{min}, r_{\beta }, \varsigma _{eff} \text { and } \beta \tau _{50}$ uniformly sampled, and $K_C, \beta _{max}, \varphi _0, P_{50}, U_{0} \text { and } P_{0}$ log-uniformly sampled (to cover their different orders of magnitude). The results were compiled in bar diagrams for both bacterial and viral load predictions.

### Considered distributions

#### Shapes of distribution considered

Different shapes of latent period distribution were simulated to investigate whether the population dynamics were sensitive to it:

- The truncated normal distribution.
- The log-normal distribution.
- The gamma distribution, often used in distributed lifespan models because of its direct equivalence to a transit compartments model when the shape parameter is a positive integer [15].

In each scenario, $sd_\tau$ was defined as the standard deviation of the latent period distribution, and $rsd_\tau = \frac{sd_\tau }{\tau _0}$ the associated relative standard deviation. Distributions with relative standard deviations ranging from 10% to 50% were considered to match ranges of variability observed experimentally [4, 16, 17].

#### Scenarios with fixed parameters

Different scenarios with a non-distributed latent period were also explored, to properly quantify the error associated with neglecting the distribution with the classical DDE model:

- Fixing the latent period to the mean of the associated distribution - referred to as "Fixed Mean".
- Fixing the latent period to the median of the associated distribution - referred to as "Fixed Median".
- Fixing the latent period to time of first observed lysis - referred to as "Fixed Early". The time of first observed lysis was computed as the time after which the number of infective centers has doubled in a single-step growth experiment (see appendix).

#### Comparison with transit compartments

**Fig. 4:**
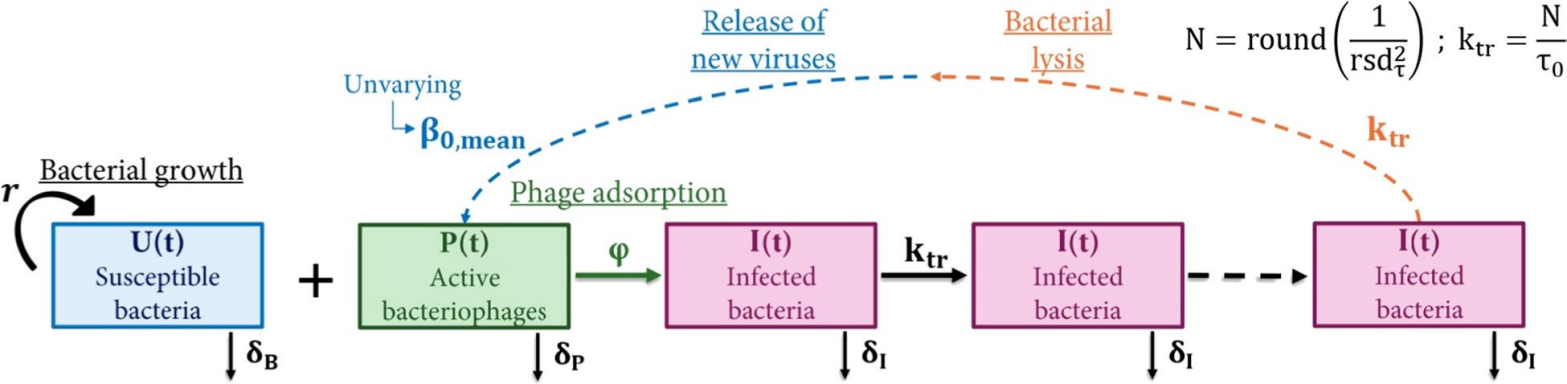
Compartmental representation of the transit compartment model, for an Erlang-distributed latent period

Transit compartments (TC) models (Fig. 4) provide a computationally efficient method to capture stochasticity in the latent period [9], equivalent to a DDDE model with Erlang-distributed latent period [15]. However, TC models do not capture the potential relationship between burst size and latent period, as presented in Eq. 5, and assume an unvarying mean burst size: $\forall \tau , \beta _0(\tau ) = \beta _{0,mean}$. Moreover, TC models do not account for $\tau _{min} \ne 0$. These represent constitutive differences with the full DDDE model, and the two are not strictly equivalent. Thus, the TC model can be seen as an approximation of the full DDDE model, and its performance was compared to the approximation with the DDE model. The number of transit compartments N and the transit rate $k_{tr}$ were defined as [15]:

9 $$\begin{aligned} N = round(\frac{1}{rsd_\tau ^2}) , k_{tr} = \frac{N}{\tau _0} \end{aligned}$$

where $\tau _0$ and $rsd_{\tau }$ are respectively the mean and the relative standard deviation of the distribution of the latent period.

### Simulation parameters

In the simulations, parameter ranges proximate to typical values reported in literature were used (Table 1), for different bacteria and phage inoculums ($U_{init}$ and $P_{init}$ respectively).

**Table 1:** Parameter ranges used in the simulations

Parameter	Simulated range	References
Bacterial growth		
$r_{max} \text (/h)$	$0.5 - 2.5$	[18]
$K_{C} \text (bact/mL)$	$10^8 - 10^{10}$	[19, 20]
Phage adsorption		
$\varphi _{0} \text (mL / cell / h)$	$10^{-9} - 10^{-7}$	[21–23]
$P_{50} \text (phage/mL)$	$\frac{r_{max}}{\varphi _0} - 10^{10}$	a.
Bacteria lysis		
$\tau _{0} \text (h)$	$0.25 - 3$	[9, 22, 24, 25]
$\tau _{min} \text (h)$	$0 - \frac{\tau _0}{2}$	N/A
Phage release		
$\beta _{mean} \text (phage/bact)$	$10 - 500$	[24–28]
$r_{\beta } \text (/h)$	$1 - 10$	[14]
$D \text (h)$	$0 - \tau _0$	b.
$\beta \tau _{50} \text (h)$	$D - 3$	c.
Decays		
$\delta _{U} \text (/h)$	$0.1 - 0.25$	[29]
$\delta _{I} \text (/h)$	$0.1 - 0.25$	d.
$\delta _{P} \text (/h)$	$0.05 - 0.15$	[30, 31]
Initial inoculums		
$U_{init} \text (bact/mL)$	$10^3 - K_C \cdot (1 - \frac{\delta _U}{r_{max}})$	e.
$P_{init} \text (phage/mL)$	$10^3 - 10^9$	N/A

a. Chosen to have a stronger maximal bactericidal effect than natural bacterial growth. b. Assuming phages have time to start replicating before lysing the bacteria. c. Taken in the range of allowed latent periods. d. Assuming similar range as uninfected bacteria decay. e. Smaller than maximal bacterial proliferation

### Numerical implementation

The models were implemented in Python 3.1, using standard Python libraries (math, numpy, scipy, matplotlib, pandas). The random seed was set to 120659 for replicability purposes.

The differential equations were solved numerically with the forward Euler method adapted to distributed delay differential equations. The Runge-Kutta 4 method was also implemented but showed no noticeable convergence benefit compared to the Euler method for the full DDDE model (see appendix). The integrals were computed as convolution sums using the history of $\varphi \cdot U \cdot P$ (see appendix). Convergence was considered achieved if dividing the step size by two induced less than a 1% difference for both the bacterial load and the viral load profiles. When convergence failed during the Monte Carlo process, a new set of parameters was generated.

To correct for truncation of the normal distribution while preserving the desired mean and standard deviation, the Newton-Raphson method was used to find appropriate parameters.

## Results

### Distributions of $\beta$ and $\varphi$ do not impact population dynamics

The analytical study demonstrated (see appendix) that the distributions of $\beta$ and $\varphi$ can be simplified in the differential equations, and only their respective means $\beta _0(\tau )$ and $\varphi (t)$ impact population dynamics. The differential Eq. 7 could thus be simplified to:

10 $$\begin{aligned} \frac{dU}{dt}(t)&= r(t) \cdot U(t) \nonumber \\&\qquad \quad - \varphi _0(t) \cdot U(t) \cdot P(t) - \delta _{U} \cdot U(t)\nonumber \\ \frac{dI}{dt}(t)&= \varphi _0(t) \cdot U(t) \cdot P(t) \\&\qquad \quad - \int _{\tau = 0}^{t} e^{- \delta _I \tau } \cdot \varphi _0(t-\tau ) \cdot U(t-\tau ) \cdot P(t-\tau ) \cdot f_{\tau }(\tau ) d\tau - \delta _I \cdot I(t) \nonumber \\ \frac{dP}{dt}(t)&= \int _{\tau = 0}^{t} \beta _{0}(\tau ) \cdot e^{- \delta _I \tau } \cdot \varphi _0(t-\tau ) \cdot U(t-\tau ) \cdot P(t-\tau ) \cdot f_{\tau }(\tau ) d\tau \nonumber \\&\qquad \qquad \qquad \qquad \qquad \qquad \quad - \varphi _0(t) \cdot (U(t) + I(t)) \cdot P(t)- \delta _P \cdot P(t) \nonumber \end{aligned}$$

where $\varphi _{0}(t)$ is the mean adsorption rate $\varphi$ at time t, and $\beta _{0}(\tau )$ is the mean burst size $\beta$ for a given latent period $\tau$. $\varphi _0(t)$ is assumed to follow Eq. 4 [13], and $\beta _0(\tau )$ to follow Eq. 5 [14]. Hereafter, Eq. 10 is used as the canonical expression for the full DDDE model.

### The shape of the distribution has negligible impact on population dynamics

**Fig. A3:**
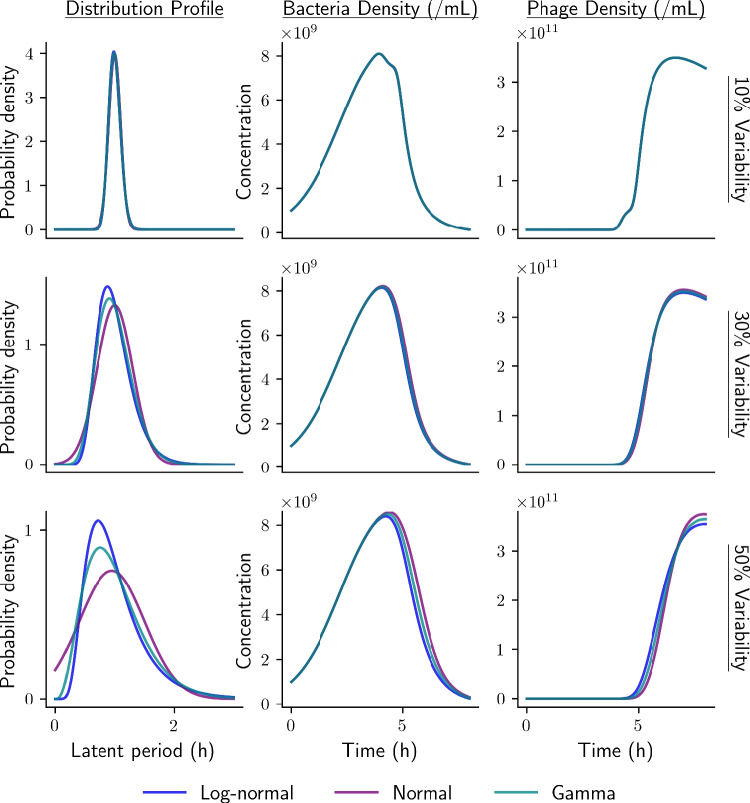
Plots of bacterial and viral load with a normal, log-normal and gamma distribution of the latent period, for an example set of parameters

Modest differences were observed visually in the bacterial and viral dynamics simulated with the lognormal, normal and gamma distributions (Fig. A3), even for wide distributions ($rsd_{\tau } = 50\%$, higher end of observed latent period variability [17])).

**Fig. 5:**
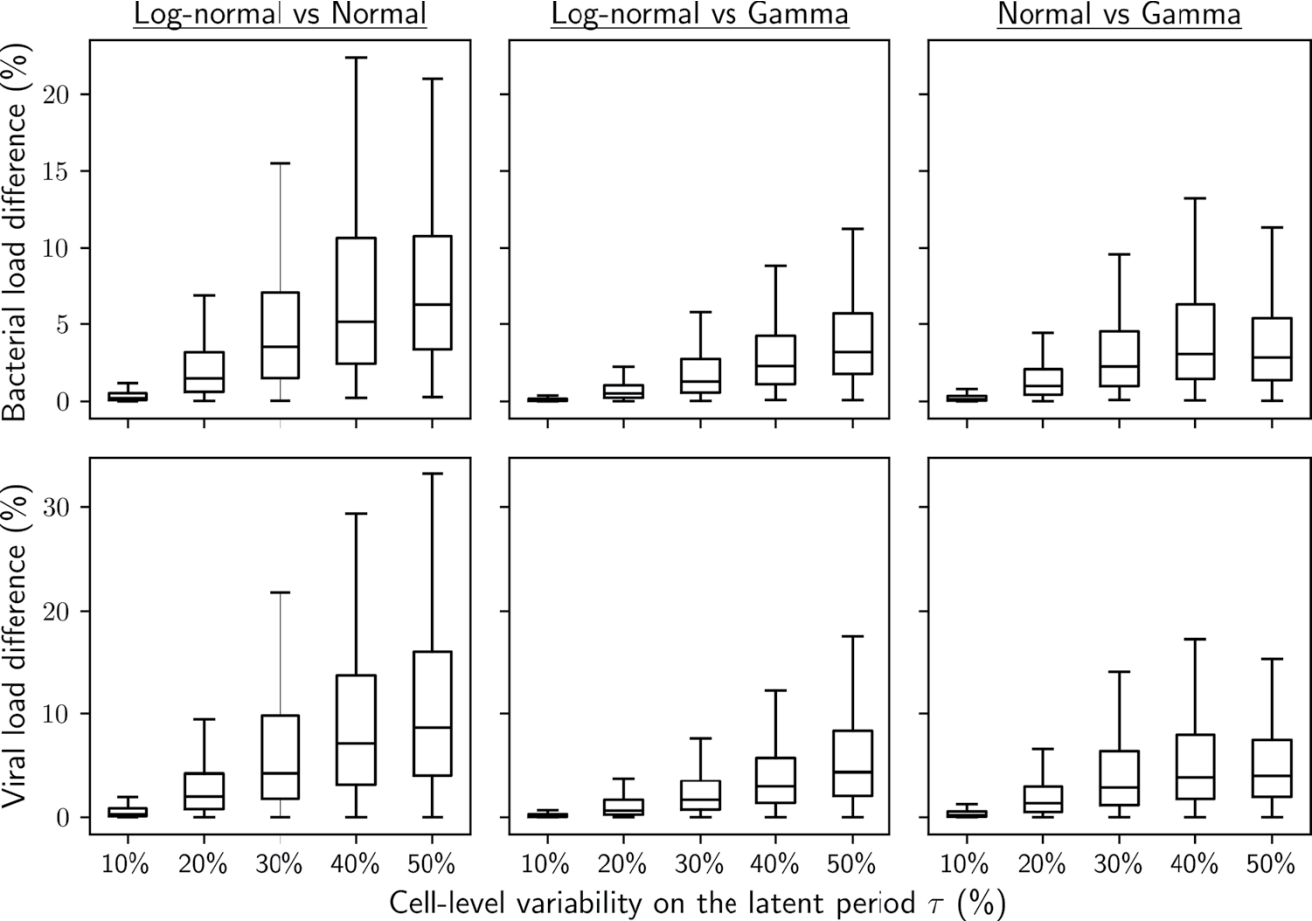
Differences in bacterial and viral load predictions between log-normally, normally and gamma distributed latent period for equal distribution width, compiled over 500 random sets of parameters

This observation was quantitatively verified over 500 simulation scenarios (Fig. 5). For $rsd_{\tau } = 50\%$, it represented less than $6.3\%$ median difference on bacterial load and $8.7\%$ median difference on viral load. Differences were observed to be larger between the log-normal and normal distributions, which is consistent with those two distribution profiles being the furthest apart (Fig. A3). In all cases, the shape of the distribution had a generally limited impact on population dynamics.

### Approximations with fixed latent period

Since the shape of the distribution was found to affect reasonably little the population dynamics, a distribution shape can be chosen arbitrarily to estimate the error induced from fixing the latent period. Here, the log-normal distribution was chosen to report the results.

**Fig. 6:**
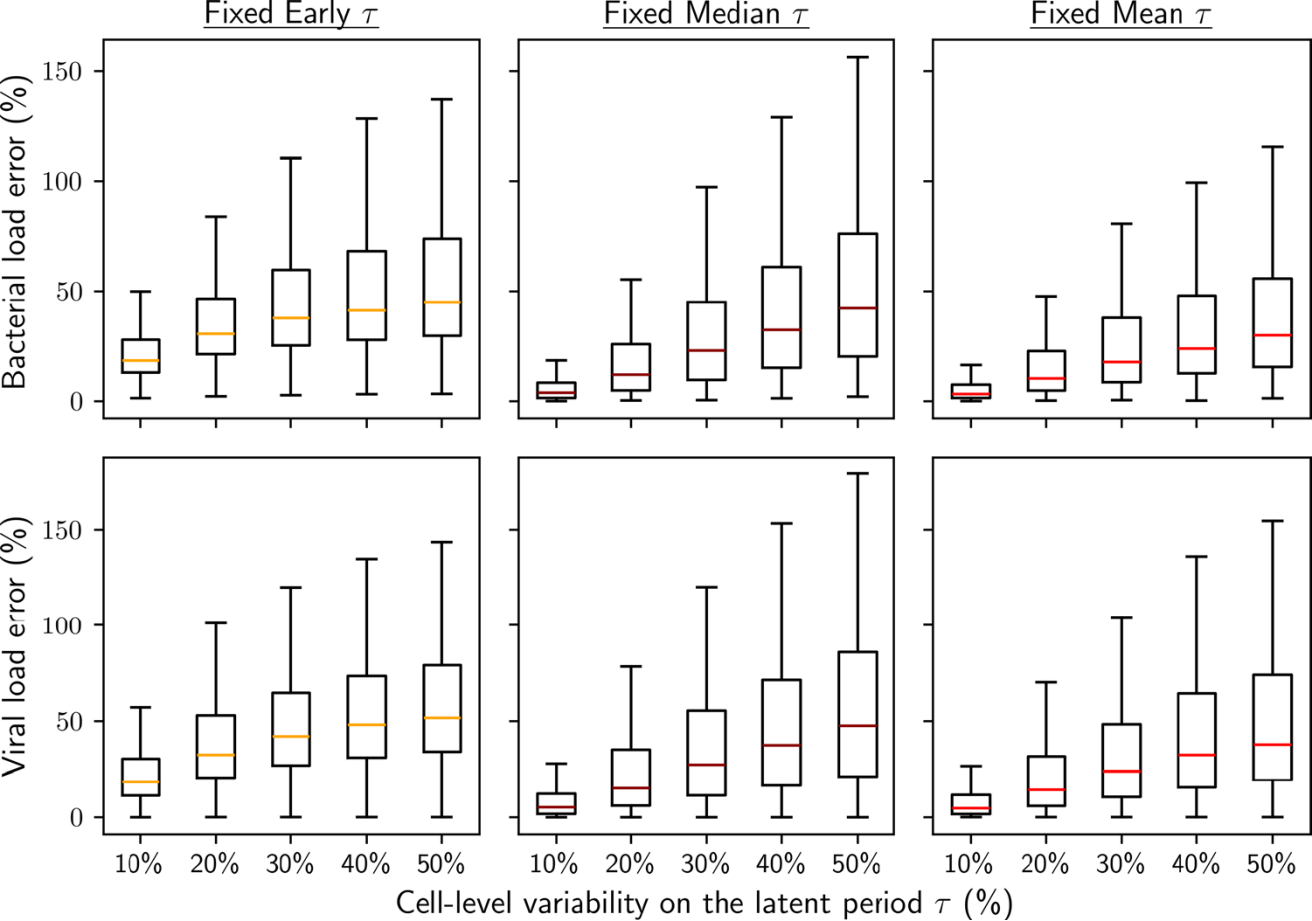
Error on bacterial and viral load predictions induced by fixing the latent period to early lysis time, to the median latent period, or to the mean latent period, compiled over 500 random sets of parameters. The reference used is a log-normally distributed latent period

**Fig. A4:**
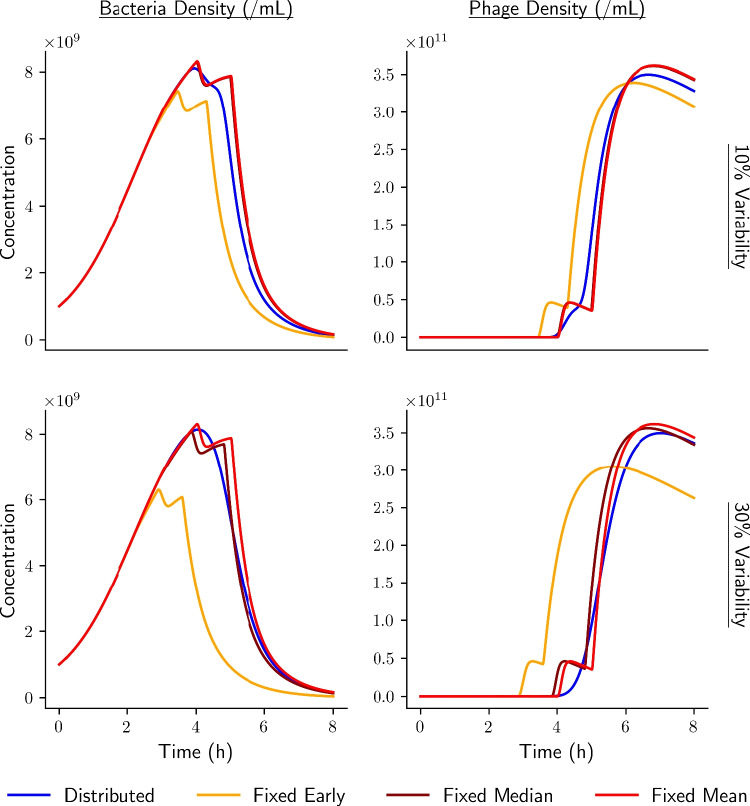
Plots of bacterial and viral load with a latent period log-normally distributed or fixed to characteristic values, for an example set of parameters

Poor concordance was observed between the reference with distributed latent period and fixing the latent period to early observed lysis (Fig. 6 and A4). Even at moderate $rsd_{\tau } = 10\%$, it already caused median bacterial and viral load prediction errors of $18.4\%$ and $18.4\%$ respectively, and these errors increased rapidly with greater variability (Fig. 6B). Therefore, fixing the latent period $\tau$ to the usually reported time to first lysis introduced a high bias in the model predictions (consistent with findings from [9, 11]).

Fixing the latent period to the median or the mean of the distribution aligned much more closely with the reference with log-normally distributed latent period. The median error on both bacterial and viral load predictions remained limited below $12.0\%$ and $15.3\%$, respectively, for a latent period variability up to $rsd_{\tau } = 20\%$, which is a typically reported range of observed variability [4, 16]. This approximation did not hold at higher latent period variability, with up to $29.8\%$ and $37.8\%$ median error respectively for $rsd_{\tau } = 50\%$ when fixing the latent period to its mean value.

### TC models do not perform better than DDE models

**Fig. 7:**
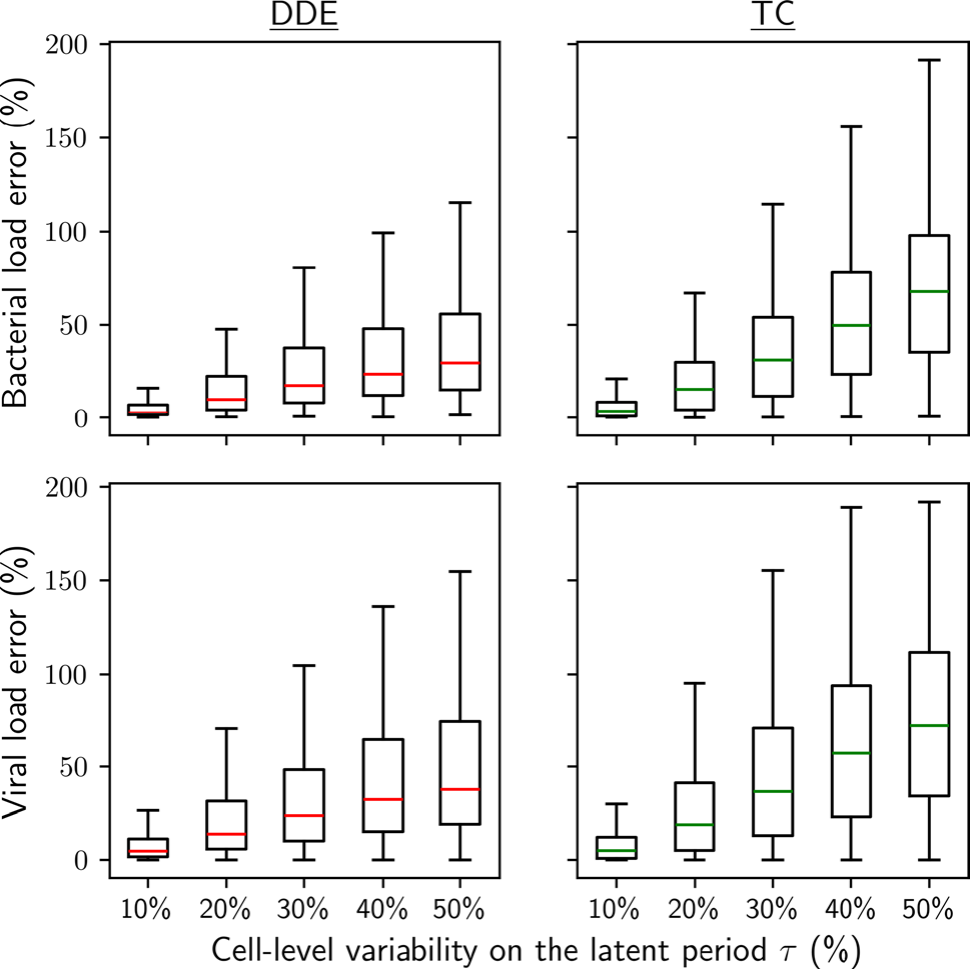
Error on bacterial and viral load predictions of the DDE and TC models relative to the full DDDE model, compiled over 500 random sets of parameters. The reference used is a log-normally distributed latent period

**Fig. A5:**
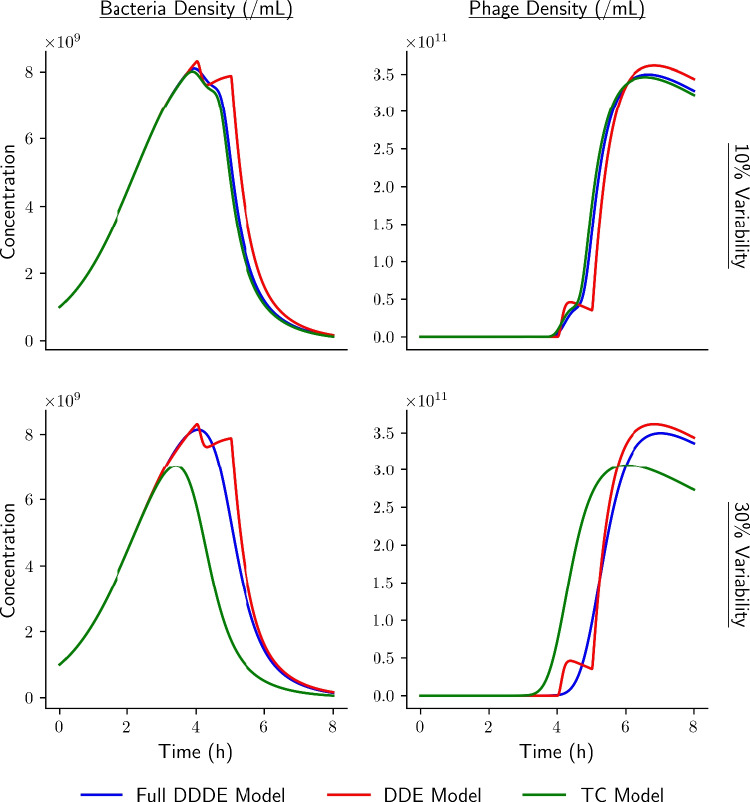
Plots of bacterial and viral load with the DDE, TC and DDDE models, for an example set of parameters

As for the approximation with the TC model, it predicted smooth dynamics similar to the full DDDE model (Fig. A5). For low latent period variability up to $rsd_{\tau } = 20\%$, this approximation resulted in reasonably low error with median of $13.5\%$ and $22.1\%$ for bacterial and viral load predictions respectively (Fig. 7). This approximation however also broke at high variability with $53.1\%$ and $63.1\%$ respective median error at $rsd_{\tau } = 50\%$. Overall, the TC model did not perform better than the Fixed Mean DDE model, which demonstrated the highest performance among the DDE models.

## Discussion

We successfully proposed a novel model structure that can account for cell-level variability as well as intracellular phage replication dynamics (Fig. 3 & Eq. 10). The random variabilities on the burst size and the adsorption rate were shown to not impact population-level dynamics, thus only the variability on the latent period and its influence on the burst size remained in the final model proposed. We then used this model structure to quantify systematically said impact to help inform more precise model building for phage dynamics modelling. A wide latent period distribution (such as the one observed for $\phi$X174 [17]) was shown to greatly influence the population-level dynamics. As for lower variability, more traditional approaches, with fixed latent period or with transit compartments, were able to predict bacterial and viral load profiles close to the ones obtained with the full model.

Since the developed model structure is a distributed delay differential equation (DDDE) model, it is computationally intensive (7s to 9s of runtime, see appendix). For narrower latent period distributions, both the fixed delay differential equation (DDE) and the transit compartments (TC) models can be close approximations of the full model. The TC model is a common usual ODE structure and can be implemented in any ODE software, while offering better run times than the full DDDE model (0.1s to 1s). However, it requires one more parameter than the DDE model, the number of transit compartments is sometimes hard to estimate, and for very narrow latent period ($rsd_\tau \le 10\%$), this number of transit compartments can be very high ($N = round(\frac{1}{rsd_\tau ^2}) \ge 100$ [15]) resulting in potentially slower run times ($\ge$ 1s). As for the DDE model, it overall performed better than the TC model, provided that the latent period should be fixed to its mean or median value rather than the time of first observed lysis, to prevent a systematic bias, while offering very efficient run times ($\approx$ 0.1s). However, DDE equations are not supported in every ODE softwares and might require some additional work to be implemented. At higher variability, the TC model introduced higher prediction errors than the DDE model in the general case. However, the former can perform exceptionally well even at high latent period variability if the burst size $\beta$ varies little with the latent period $\tau$, $\beta _0(\tau ) \approx constant$, for instance if we can neglect the dynamics relative to the intracellular viral replication (see appendix). As such, a priori knowledge of expected latent period variability and intracellular dynamics can greatly help inform model selection. In practice, estimating phage parameters from population dynamics in the DDE and TC models may result in lower errors than previously described. However, this may introduce bias on the other parameters (burst size, adsorption rate) to compensate for the latent period misspecification. An extension of this work could thus explore how well those two models can fit profiles simulated with the full DDDE model, and whether such models remain reliable for extrapolation beyond the range used for the parameter estimation.

The approach proposed here remains theoretical, based on a semi-mechanistic structural model. Despite using model parameters in typically observed ranges, the parameter combinations simulated might not always correspond to real bacteriophages, and we only compared a few usual distributions for the distributed parameters. Given available experimental data [4, 16], distributions were not expected to differ greatly from a normal distribution. As such only normal, log-normal and gamma distributions were considered in this work. Yet, other distributions, especially less-standard distributions, such as bimodal distributions, might result in different trends. The results discussed here should thus be further validated through experimental studies. While precise measurement of cell-level variability can require labor-intensive single-cell experiments and cannot be easily applied to wider pharmacological applications, population-level studies can still provide valuable insights into the underlying distribution, as demonstrated through single-step growth curves. Tracking bacterial load and viral load over a single round of infection could allow us to identify latent period distribution and the correlation between burst size and latent period, and assess the concordance of the developed model with real profiles.

This work can also provide insight on model selection in different contexts. For simulation purposes, run time is often a less critical criterion and the full DDDE model may be preferred since it is expected to capture the dynamics more mechanistically. Measuring cell-level heterogeneity, to properly inform the full DDDE model, can however remain challenging experimentally, and different assumptions and translation work from other phages might be required. For estimation purposes, both the DDE and the TC model offer a much more practical run time. Given that phages generally exhibit low variability in latent period [4, 16], it may be advisable to first try using the DDE model, which requires the least parameters, in absence of prior knowledge regarding the variability. In that case, it is preferable to either directly estimate a latent period from population dynamics, if feasible, or to fix it based on an experimentally determined median or mean value. A TC model can also be tested, especially in software with advanced traditional ODE solvers that might not be compatible with DDE models. In this case, the number of transit compartments can either be fixed to an arbitrary value higher than N = 10, to account for the fact that typical rsd is less than $30\%$, or estimated. Conversely, if experiments show evidence of strong latent period variability, i.e., a gradual single-step growth curve, or if the previous two models fail to capture the profile accurately, more accurate distributed approaches may still be necessary. Some approaches relying on partial differential equations can for instance be used to allow for a thorough description of aging dynamics of the infected bacteria [32, 33], but those are highly computationally intensive and difficult to implement in some modeling softwares often used in pharmacometrics, such as NONMEM. The full DDDE model with intracellular replication dynamics could offer a good in-between, that efficiently captures the infection age of infected cells to track cell-level dynamics. We are also currently working on an ODE approximation of this approach to further facilitate integration into any ODE solver, improve significantly run time, and allow for a potentially broader use in the field.

In conclusion, the classical fixed delay differential equation model should thus be used in first intention for phage modelling. However, if prior knowledge of latent period variability is available or if the fixed delay approach fails, a distributed approach is warranted and the full DDDE model may be the preferred option in most cases.

## Supplementary Information

Below is the link to the electronic supplementary material.

Supplementary file 1 (zip 18 KB)

## Figures and Tables

**Fig. A1:**
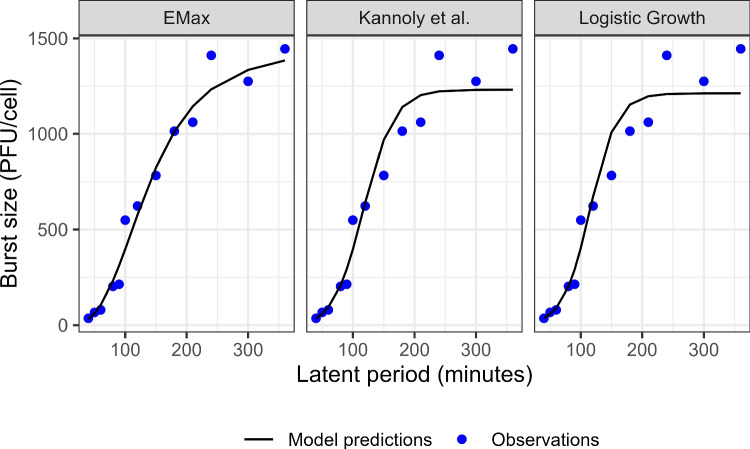
Fits of $\tau \rightarrow \beta (\tau )$ with different phenomenological models, observations were digitized from [14]

**Fig. A2:**
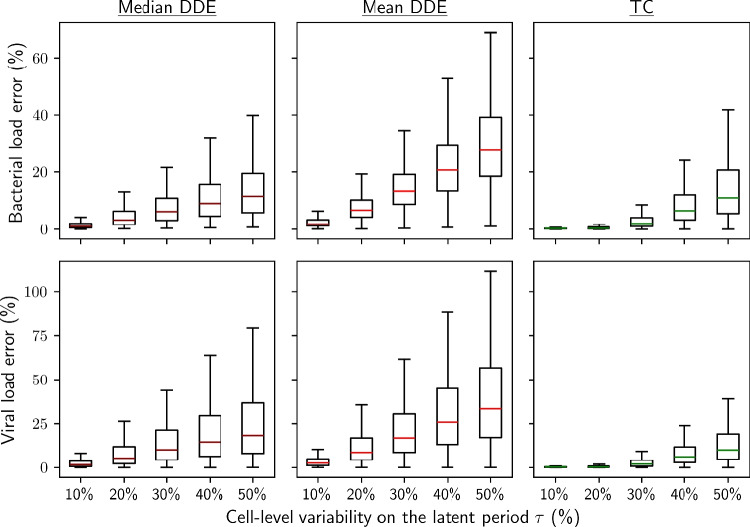
Error on bacterial and viral load predictions of the DDE and TC models relative to the full DDDE model, compiled over 100 random sets of parameters, with $\beta _0(\tau ) = cst$

## Data Availability

Numerical models were developed and model predictions were generated in this study. The model code has been submitted as supplementary material.

## Appendices

### Normalization of $\beta (\tau )$

The population mean burst size, $\beta _{0,mean} = \int _0^{+\infty } \beta _0(\tau )f_\tau (\tau )d\tau$, is the parameter typically determined from single-step growth curves [8]. Therefore, $\beta _{max}$ in Eq. 5 was re-expressed using the following transformation:

A1 $$\begin{aligned} \beta _{max} = \frac{\beta _{0,mean}}{\frac{\beta _{0,mean}}{\beta _{max}}} = \frac{\beta _{0,mean}}{\frac{\int _{\tau = \tau _{min}}^{+\infty } \beta _0(\tau ) \cdot f_\tau (\tau ) \cdot d\tau }{\beta _{max}}} \end{aligned}$$

Using Eq. 5 to express $\beta _0(\tau )$, we get:

A2 $$\begin{aligned} \beta _{max} = \frac{\beta _{0,mean}}{\frac{\int _{\tau = \tau _{min}}^{+\infty } \frac{\beta _{max} e^{r_\beta (\tau - D)} - 1}{e^{r_\beta (\beta \tau _{50} - D)} + e^{r_\beta (\tau - D)} - 2} \cdot f_\tau (\tau ) \cdot d\tau }{\beta _{max}}} \end{aligned}$$

$\beta _{max}$ cancels out in the denominator, which gives Eq. 6:

$$\begin{aligned} \beta _{max} = \frac{\beta _{0, mean}}{\int _{\tau = \tau _{min}}^{+\infty } \frac{e^{r_\beta (\tau - D)} - 1}{e^{r_\beta (\beta \tau _{50} - D)} + e^{r_\beta (\tau - D)} - 2} f_\tau (\tau ) d\tau } \end{aligned}$$

### Analytical reduction of the full DDDE framework

Equation 7 describes naively population dynamics with distributed phage parameters:

$$\begin{aligned} \frac{dU}{dt}(t)&= r(t) \cdot U(t) \\&\qquad \quad - \int _{\varphi = 0}^{+ \infty } \varphi \cdot U(t) \cdot P(t) \cdot f_{\varphi | t}(\varphi | t) \cdot d\varphi - \delta _{U} \cdot U(t)\\ \frac{dI}{dt}(t)&= \int _{\varphi = 0}^{+ \infty } \varphi \cdot U(t) \cdot P(t) \cdot f_{\varphi | t}(\varphi | t) \cdot d\varphi \\&\quad - \int _{\tau = 0}^{t} \int _{\varphi = 0}^{+ \infty } e^{- \delta _I \tau } \cdot \varphi \cdot U(t-\tau ) \cdot P(t-\tau ) \\&\qquad \,\,\cdot f_{\varphi | t}(\varphi | t - \tau ) \cdot f_{\tau }(\tau ) \cdot d\varphi \cdot d\tau - \delta _I \cdot I(t)\\ \frac{dP}{dt}(t)&= \int _{\tau = 0}^{t} \int _{\varphi = 0}^{+ \infty } \int _{\beta = 0}^{+ \infty } \beta \cdot e^{- \delta _I \tau } \cdot \varphi \cdot U(t-\tau ) \cdot P(t-\tau ) \\&\qquad \qquad \quad \cdot f_{\beta | \tau }(\beta | \tau ) \cdot f_{\varphi | t}(\varphi | t-\tau ) \cdot f_{\tau }(\tau ) \cdot d\beta \cdot d\varphi \cdot d\tau \\&\qquad \quad - \int _{\varphi = 0}^{+ \infty } \varphi \cdot (U(t) + I(t)) \cdot P(t) \cdot f_{\varphi | t}(\varphi | t) d\varphi - \delta _P \cdot P(t) \end{aligned}$$

Reorganizing the terms inside the integrals gives:

A3 $$\begin{aligned} \frac{dU}{dt}(t)&= r(t) \cdot U(t) \nonumber \\&\qquad \quad - U(t) \cdot P(t) \cdot [\int _{\varphi = 0}^{+ \infty } \varphi \cdot f_{\varphi | t}(\varphi | t) \cdot d\varphi ] - \delta _{U} \cdot U(t)\nonumber \\ \frac{dI}{dt}(t)&= U(t) \cdot P(t) \cdot [\int _{\varphi = 0}^{+ \infty } \varphi \cdot f_{\varphi | t}(\varphi | t) \cdot d\varphi ] \\&\qquad \qquad \qquad - \int _{\tau = 0}^{t} e^{- \delta _I \tau } \cdot U(t-\tau ) \cdot P(t-\tau ) \nonumber \\&\qquad \cdot [\int _{\varphi = 0}^{+ \infty } \varphi \cdot f_{\varphi | t}(\varphi | t - \tau ) \cdot d\varphi ] \cdot f_{\tau }(\tau ) \cdot d\tau - \delta _I \cdot I(t) \nonumber \\ \frac{dP}{dt}(t)&= \int _{\tau = 0}^{t} e^{- \delta _I \tau } \cdot U(t-\tau ) \cdot P(t-\tau ) \nonumber \\&\qquad \cdot [\int _{\varphi = 0}^{+ \infty } [\int _{\beta = 0}^{+ \infty } \beta \cdot f_{\beta | \tau }(\beta | \tau ) \cdot d\beta ] \cdot \varphi \cdot f_{\varphi | t}(\varphi | t-\tau ) \cdot f_{\tau }(\tau ) \cdot d\varphi ] \cdot d\tau \nonumber \\&\qquad \qquad \qquad - (U(t) + I(t)) \cdot P(t) \cdot [\int _{\varphi = 0}^{+ \infty } \varphi \cdot f_{\varphi | t}(\varphi | t) d\varphi ]- \delta _P \cdot P(t)\nonumber \end{aligned}$$

Per definition, $[\int _{\beta = 0}^{+ \infty } \beta \cdot f_{\beta | \tau }(\beta | \tau ) \cdot d\beta ] = \beta _0(\tau )$ is the mean burst size $\beta$ for a given latent period $\tau$ and is independent of $\varphi$, hence:

A4 $$\begin{aligned} \frac{dU}{dt}(t)&= r(t) \cdot U(t) \nonumber \\&\qquad \quad - U(t) \cdot P(t) \cdot [\int _{\varphi = 0}^{+ \infty } \varphi \cdot f_{\varphi | t}(\varphi | t) \cdot d\varphi ] - \delta _{U} \cdot U(t)\nonumber \\ \frac{dI}{dt}(t)&= U(t) \cdot P(t) \cdot [\int _{\varphi = 0}^{+ \infty } \varphi \cdot f_{\varphi | t}(\varphi | t) \cdot d\varphi ] \\&\qquad \qquad \qquad \quad - \int _{\tau = 0}^{t} e^{- \delta _I \tau } \cdot U(t-\tau ) \cdot P(t-\tau ) \nonumber \\&\qquad \quad \cdot [\int _{\varphi = 0}^{+ \infty } \varphi \cdot f_{\varphi | t}(\varphi | t - \tau ) \cdot d\varphi ] \cdot f_{\tau }(\tau ) \cdot d\tau - \delta _I \cdot I(t)\nonumber \\ \frac{dP}{dt}(t)&= \int _{\tau = 0}^{t} e^{- \delta _I \tau } \cdot U(t-\tau ) \cdot P(t-\tau ) \cdot \beta _0(\tau ) \nonumber \\&\qquad \qquad \qquad \cdot [\int _{\varphi = 0}^{+ \infty } \varphi \cdot f_{\varphi | t}(\varphi | t-\tau ) \cdot f_{\tau }(\tau ) \cdot d\varphi ] \cdot d\tau \nonumber \\&\qquad \quad - (U(t) + I(t)) \cdot P(t) \cdot [\int _{\varphi = 0}^{+ \infty } \varphi \cdot f_{\varphi | t}(\varphi | t) d\varphi ]- \delta _P \cdot P(t)\nonumber \end{aligned}$$

Also per definition, for any time $\varsigma$, $[\int _{\varphi = 0}^{+ \infty } \varphi \cdot f_{\varphi | \varsigma }(\varphi | \varsigma ) \cdot d\varphi ] = \varphi _0(\varsigma )$ is the mean adsorption rate $\varphi$ at time $\varsigma$. By applying this for times $\varsigma = t$ and $\varsigma = t - \tau$, we get the simplified Eq. 10:

$$\begin{aligned} \frac{dU}{dt}(t)&= r(t) \cdot U(t) \\&\qquad \quad - \varphi _0(t) \cdot U(t) \cdot P(t) - \delta _{U} \cdot U(t)\\ \frac{dI}{dt}(t)&= \varphi _0(t) \cdot U(t) \cdot P(t) \\&\qquad \qquad - \int _{\tau = 0}^{t} e^{- \delta _I \tau } \cdot \varphi _0(t-\tau ) \cdot U(t-\tau ) \cdot P(t-\tau ) \cdot f_{\tau }(\tau ) \cdot d\tau - \delta _I \cdot I(t)\\ \frac{dP}{dt}(t)&= \int _{\tau = 0}^{t} \beta _{0}(\tau ) \cdot e^{- \delta _I \tau } \cdot \varphi _0(t-\tau ) \cdot U(t-\tau ) \cdot P(t-\tau ) \cdot f_{\tau }(\tau ) \cdot d\tau \\&\qquad \qquad \qquad \qquad \qquad \qquad \qquad - \varphi _0(t) \cdot (U(t) + I(t)) \cdot P(t)- \delta _P \cdot P(t) \end{aligned}$$

### Additional information on the numerical implementation of the DDDE system

The DDDE system was solved using the forward Euler method. To compute the integrals, the history of the infection term $contam(t) = (\varphi_0 \cdot U \cdot P)(t)$ was kept in memory during each solver step. This history was used to compute two convolution sums:

- $$\begin{aligned} It_I(t) = \int _{\tau = 0}^{t} e^{- \delta _I \tau } \cdot (\varphi _0 \cdot U \cdot P)(t-\tau ) \cdot f_{\tau }(\tau ) \cdot d\tau \end{aligned}$$ is computed as $It_I(t) = (g_{lysis} \circledast contam)(t)$ where $g_{lysis}(\tau ) = e^{-\delta _I \tau } \cdot f_{\tau }(\tau )$
- $$\begin{aligned} It_P(t) = \int _{\tau = 0}^{t} \beta _0(\tau ) \cdot e^{- \delta _I \tau } \cdot (\varphi _0 \cdot U \cdot P)(t-\tau ) \cdot f_{\tau }(\tau ) \cdot d\tau \end{aligned}$$ is computed as $It_P(t) = (g_{release} \circledast contam)(t)$ where $g_{release}(\tau ) = \beta _0(\tau ) \cdot e^{-\delta _I \tau } \cdot f_{\tau }(\tau )$

Both $g_{lysis}$ and $g_{release}$ do not depend on state variables and are thus pre-computed into an array outside of the ODE solver. Two versions of the convolution sums were implemented, one over the complete history and one only up to $\tau = 5 \times \tau _0$ to reduce computational burden. No significant difference was observed between the two approaches.

### Further information regarding the fixed early latent period

The fixed early latent period was defined as the latent period after which, after simultaneous infection with phages and in absence of bacterial elimination, the amount of free phages released by bacterial lysis is twice the initial phage inoculum. Formally $\tau _{early}$ is defined as the latent period that satisfies Eq. A5.

A5 $$\begin{aligned} \int _0^{\tau _{early}} \beta _0(\tau ) \cdot f\tau (\tau ) \cdot d\tau = 2 \end{aligned}$$

### Other dependency profiles between $\tau$ and $\beta$

In this paper, Eq. 5 was used to describe the relationship between the latent period $\tau$ and the burst size $\beta$. However, other models can also accurately capture the dynamics observed by Kannoly et al. [14] (Fig. A1).

The model structure proposed in this work is flexible and can be adapted with any expression for $\beta _0(\tau )$. In particular, a closed-form expression is not required as one can numerically solve for $\beta _0(\tau )$ and plug the approximate solution into the full DDDE model.

### Results with a fixed burst size

The analysis proposed in this work was repeated with different expressions of $\beta _0(\tau )$. The case $\beta _0(\tau ) = cst$ was especially of interest, as this is a common assumption in classical viral dynamics models.

As expected, for $\beta _0(\tau ) = cst$, the TC model performed exceptionally well even at high variability on the latent period, with $11.1 \%$ and $10.1 \%$ median error for bacterial concentration and viral concentration respectively for $rsd = 50 \%$. Fixing the latent period to its median value also offered a very good approximation in this case, with median errors of $11.6\%$ and $18.4\%$ respectively (Fig. A2).

### Run time comparison of the full DDDE, DDE and TC models

The full DDDE, the DDE and the TC models were ran on an AMD Ryzen 5 PRO 7540 CPU using the forward Euler method to compare their respective run times. Said run times were measured during the 500 simulations used for comparing the approximation with the TC and the DDE models (Figs. 7).

The DDE model offered the fastest mean run time with the forward Euler solver, over 70 times faster than the full DDDE model (Table A1). The TC model gave similar run times as the DDE model for high rsd but performed slower for narrower distributions, because it requires quadratically more transit compartments when rsd decreases ($N = round(1/rsd_{\tau }^2)$). In practice, both models run times can be further optimized with more advanced solvers, especially the TC model being a traditional ODE-based model.

**Table A1:** Comparative mean run times for the different models at different rsd

rsd	10 %	20 %	30 %	40 %	50 %
Full DDDE	8.3s	8.3s	7.9s	7.5s	7.6s
DDE	0.1s	0.1s	0.1s	0.1s	0.1s
TC	1.1s	0.3s	0.2s	0.1s	0.1s

### Convergence of the Euler and Runge-Kutta solvers

Runge-Kutta 4 is another commonly used ODE solver, usually offering better convergence than the simple forward Euler method. Both solvers were thus implemented for the full DDDE model, and their perforamce was compared over 300 simulated scenarios. The scenarios were divided between normal, lognormal and gamma distributions, for rsd levels ranging from 10% to 50%, and with parameters randomly sampled as explained in the methods. The numerical convergence criterion was tested for the forward Euler solver and the Runge-Kutta (RK4) solver for different step sizes, to assess if the RK4 solver improved significantly convergence.

Overall, the Runge-Kutta solver did not show better achievement of the convergence criterion for a same step size, while requiring longer computation (Table A2). As such, the forward Euler solver was used as our default solver in this study.

**Table A2:** % of simulations reaching the convergence criterion for different step sizes, and mean run time of converging runs

Solver	Step size				Mean runtime
	2e-2	1e-2	5e-3	2.5e-3	
Euler method	54.0%	75.0%	92.3%	98.7%	8.2s
RK4 method	62.3%	82.3%	93,7%	97.7%	20.0s
